# Getting Started with Taiji: Investigating Students Expectations and Teachers Appraisals of Taiji Beginners Courses

**DOI:** 10.1155/2012/595710

**Published:** 2012-11-13

**Authors:** Marko Nedeljkovic, Christina Bürgler, Petra H. Wirtz, Roland Seiler, Konrad M. Streitberger, Brigitte Ausfeld-Hafter

**Affiliations:** ^1^Institute of Complementary Medicine KIKOM, University of Bern, Imhoof-Pavillon, Inselspital, 3010 Bern, Switzerland; ^2^Department of Psychology, University of Bern, Biological and Health Psychology, Alpeneggstraße 22, 3012 Bern, Switzerland; ^3^Institute of Sport Science, University of Bern, Alpeneggstraße 22, 3012 Bern, Switzerland; ^4^Department of Anesthesiology and Pain Therapy, University Hospital Bern, Inselspital, 3010 Bern, Switzerland

## Abstract

In recent years, Taiji has been frequently investigated and considered as a stress management intervention. Although health care providers' appraisals and consumers' expectations are regarded as essential for treatment outcome, little attention has been drawn to this issue in Taiji research. In our study we have conducted two surveys to explore beginners' (*n* = 74) expectations and teachers' (*n* = 136) appraisals of their Taiji courses in general as well as more particularly related to stress management. Qualitative data analysis revealed that beginners mainly expected to learn a new method that is applicable in their daily life to foster peace of mind and to enhance their stress management. Congruently moderate-to-high improvements in stress management have also been found in quantitative analysis, whereby a lower educational level predicted higher expectations (*P* = 0.016). Taiji-teachers stated body- and mind-related benefits most frequently and appraised moderate-to-high improvements in stress management. Higher appraisals were predicted by a shorter teaching experience (*P* = 0.024). Our results inform about beginners' expectations and teachers' appraisals related to a Taiji-beginners course and highlight the role of educational background and teaching experience in shaping stress-management-related beginners' expectations and teachers' appraisals.

## 1. Introduction

In the recent past, the interest in mind-body practices for health promotion and stress management has considerably increased in the general and clinical population as well as in the scientific community [1–5]. In particular, Taiji (or T'ai Chi, T'ai Chi Chuan, Taijiquan), a mind-body practice originating from China, became more popular in western countries in the last decades [6–8]. Taiji is defined by Wayne and Kaptchuk [6] as “an exercise based on slow intentional movements, often coordinated with breathing and imagery, which aims to strengthen and relax the physical body- and mind, enhance the natural flow of what the Chinese call *qi* (…, life energy), and improve health, personal development, and self-defense” (page 96). In fact, numerous clinical trials and systematic reviews examined the effectiveness of Taiji for various health conditions, underlining its preventive and therapeutic value, for example, for fall prevention [9–12], for treatment of chronic diseases [9, 13–16], and for improvement of mental health [9, 10, 17], where a particularly growing body of evidence is supporting the beneficial effects of Taiji practice on stress management [17–22]. However, hitherto only a few studies have been published explicitly investigating the underlying modes of action of Taiji [6, 23, 24]. Taiji is regarded as a complex intervention, comprising multiple components of which each may have independent and synergistic therapeutic value. Two of these components are students' expectations and teachers' attitudes [6]. 

As shown in previous research, treatment expectations of health care consumers may influence treatment outcome; in particular higher treatment expectations have been repeatedly found to be associated with better treatment results [25–29]. The impact of health care practitioners' expectations on treatment results has also been documented [30–32] as well as the crucial importance of the match of treatment-related appraisals and expectations for an outcome enhancing working alliance [33–36].

Even though the above-mentioned findings underline the relevance of expectancies and appraisals on treatment results, to date studies exploring this issue in the field of Taiji and other mind-body practices are scarce. We have found an early Taiji study, where the enhancement of mood after Taiji practice has been partially explained by a higher expectation of a positive outcome (i.e., mood enhancement) in the Taiji group [37]. Although the need for further research into the role of participants' motivation in stress management practices such as Taiji has been highlighted [37], only one qualitative study assessed treatment-related expectations of Qigong beginners [38]. The findings of this study suggest that Qigong beginners with no further specified health status mainly expect improvement of their health condition and relaxation as well as professionalism, provision of information, and empathy from the teachers [38]. To the best of our knowledge, beginners' expectations and teachers' appraisals regarding the benefits of their Taiji courses have not yet been investigated.

Based on the relevance of treatment-related expectations and appraisals for treatment outcome, an increased awareness and knowledge about beginners' expectations and teachers' appraisals of their Taiji-beginners courses may have an impact on treatment outcomes in Taiji interventions. Therefore, the aim of our present study was to explore beginners' expectations and teachers' appraisals of their Taiji-beginners courses.

## 2. Methods

### 2.1. Study Design

We have conducted two surveys, one among Taiji-beginners in the area of Bern and one with Taiji teachers in the German speaking area of Switzerland including the Bern area. The survey of Taiji-beginners was nested within a trial examining psychobiological effects of Taiji on psychosocial stress reactivity [22] and was formally approved by the ethics committee of the Canton of Bern, Switzerland. For the survey of the Taiji teachers, no approval of the ethics committee was required. However, participants' information about the study and the voluntary nature of survey participation, and data protection were handled alike. 

### 2.2. Selection of Subjects

In the first survey, healthy Taiji-beginners were recruited through announcements on pin boards and on the websites of the University of Bern and the University Hospital in Bern. Eligible participants had to be between 18 and 50 years old and fluent in German. Exclusion criteria are reported elsewhere in detail [22]. All participants who completed baseline examination were included in this study.

For the second survey, we identified electronically registered Taiji teachers in the German-speaking part of Switzerland by conducting an Internet search in November 2010 using the Google search engine. All of them were invited to participate in an online survey by e-mail. Participants had to be fluent in German and actively engaged as Taiji teachers.

### 2.3. Data Collection

Both study groups participated in an online survey. After assessing sociodemographic data (for all participants: gender, age, and occupation status; for Taiji-beginners only: education level; for Taiji teachers only: years of Taiji practice experience and years of Taiji-teaching experience) the first question asked to Taiji-beginners aimed to assess their general expectations and was “What are your expectations towards the upcoming Taiji course (= two lessons per week during 3 months)?” Comparably, the Taiji teachers were asked to answer the open question “Which benefits can a newbie expect from a Taiji-beginners course (= two lessons per week during 3 months)?” also by writing down their narrative responses. 

To assess beginners' expectations and teachers' appraisals related to changes in stress management in response to regular Taiji practice, all study participants were additionally asked to rate 12 statements expressing Taiji-induced changes in stress coping (6 items) and resource activation (6 items), by indicating the degree of their agreement on a 6-point Likert scale ranging from 1 (strongly disagree) to 6 (strongly agree). To avoid priming effects, these items were presented on a new screen page. The full self-developed questionnaire is shown in the Appendix. We defined the sum of all rating scores as an index representing expected or appraised changes in overall stress management induced by regular Taiji practice. This stress management index with a theoretical score range from 12 to 72 has a high internal consistency in Taiji-beginners (Cronbach's *α* = 0.89), as well as in Taiji teachers (Cronbach's *α* = 0.94). Construct validity was estimated by pooling data of both study groups and calculating a principal component factor analysis across all 12 items. An analysis of the eigenvalues using scree test [39] resulted in a one general factor solution (eigenvalue = 6.5) with 54.4% explanation of variance. The item loadings on the general factor ranged from 0.45 to 0.83. 

### 2.4. Data Analysis

Data analysis was conducted by using SPSS (version 18) statistical software package for Macintosh (IBM SPSS Statistics. Somers, NY, USA). Sociodemographic characteristics of Taiji-beginners and Taiji teachers were analyzed by using descriptive statistics. Unless indicated, all results are presented as mean ± standard deviation (SD).

Narrative questionnaire data were systematically prepared and analyzed by using a qualitative and quantitative approach [40]. In the qualitative approach, each analytical step has been conducted independently by two authors (MN and CB). After each step, results were compared and differences were discussed until consensus was found. In a first step, each narrative response was screened to detect and mark all analytical units (e.g., beginners' expectations, resp., teachers' perceived benefits). In a second step, those analytical units lacking in terminological clarity were explicitly stated. Afterwards, all analytical units were reduced to short paraphrases, comprehensible independent from its originally embedded context. In a next step, we conducted a content analysis of about 50% of all analytical units and inductively generated thematic categories. The suitability of these categories was tested by classifying the remaining 50% of all analytical units. After amending the initially defined categories, we reclassified all analytical units. Finally, we thematically captured the final categories into main categories. Both the main and the subcategories were quantitatively described by indicating the frequency of mentions in absolute and percentage values. 

In explorative data analysis, we compared beginners' general expectations with teachers' general appraisals related to Taiji-beginners courses by examining group differences of frequency values in each main category using *χ* ^2^ tests. 

In the quantitative approach, we conducted explorative comparisons of stress-management-related to beginners' expectations and teachers' appraisals using a *t*-test for independent samples. Prior to *t*-test calculation, normal distribution of data and homogeneity of variance were verified by the Kolmogorov-Smirnov test and the Levene test. All analyses were two tailed, with the level of significance set at *P* ≤ 0.05 and the level of borderline significance set at *P* ≤ 0.10.

For Taiji-beginners, we calculated a hierarchical linear regression analysis to examine the predictive value of the independent variables “age,” “gender,” and “education level” on the expected changes in overall stress-management-related to regular Taiji practice (dependent variable). Similarly, we computed a hierarchical linear regression analysis in the group of Taiji teachers to investigate the potential role of the variables “age,” “gender,” and “teaching experience” as independent predictors of their appraised changes in Taiji-beginners' overall stress management due to regular Taiji practice (dependent variable).

## 3. Results 

### 3.1. Group Characteristics

Of the 112 initially registered applicants for a Taiji-beginners course, 74 subjects completed baseline examination and met the inclusion but none of the exclusion criteria. There was no missing data in this group.

Of the 355 invited Taiji teachers, 24 had no valid e-mail address and could not have been reached otherwise, 19 were offering Qigong but no Taiji classes, 10 were not teaching anymore, and 10 were registered twice. Of the remaining 292 potentially eligible Taiji teachers, 136 (47%) completed the survey. Stress-management-related appraisals were missing from three Taiji teachers. An overview of sociodemographic characteristics of both study groups is presented in Table 1.

### 3.2. Beginners' Expectations towards Their Taiji Course

#### 3.2.1. Qualitative Analyses

**Figure 1:**
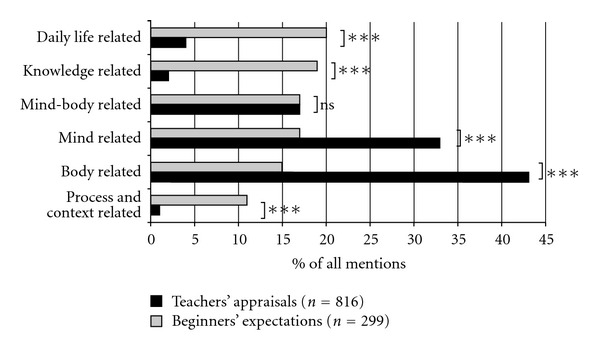
Comparison of aggregated beginners' expectations and teachers' appraisals; ns = not significant; *** = *P* < 0.001.

Analyzing Taiji-beginners' answers on the first question assessing course expectations in general, a total of 299 expectations (mean 4.04 ± 1.84) were stated. As shown in Figure 1, beginners mentioned daily-life-related expectations (comprising 20% of all expectations mentioned) approximately as frequently as knowledge related (19%), mind-related (17%), and mind-body-related expectations (17%), while body-related (15%) and process and context-related expectations (11%) were mentioned less frequently. 

With respect to frequencies of expectations as represented in the subcategories, “get to know Taiji in general” was mentioned by 57% of all Taiji-beginners followed by “improvement of stress management” (41%) and “increase of internal balance and peace of mind” (32%). 27% of all beginners also expected “transferability of course content into daily life” and an “increase of body awareness”. The expectation “increase of relaxation” was mentioned by 26% of the course applicants. Complete results are shown in Table 2.

#### 3.2.2. Quantitative Analyses

From regular Taiji practice (i.e., one hour twice a week during three months) beginners expected a moderate-to-high improvement of their stress management (mean 53.50 ± 9.56; range = 12 to 72). Regression analysis revealed that a lower education level significantly predicted higher improvements in the successful management of stress (*β* = − 0.29; *P* = 0.016), whereas age and gender did not (see Table 3). The whole model explained 9.1% of total variance in beginners expected stress-management-related changes (*R* ^2^ = 0.091; *R* ^2^corr = 0.052; *F* = 2.32, df = 3/73, *P* = 0.083) with 8.0% explained by “educational level” (*P* = 0.016).

### 3.3. Teachers' Appraisals of Their Taiji Courses

#### 3.3.1. Qualitative Analyses

A total of 816 general appraisals (mean = 6.00 ± 2.76) were stated by the Taiji teachers in their answers to the initial question assessing potential benefits a Taiji-beginners course may offer to newbies. As shown in Figure 1, 76% of all appraisals belonged to the main categories of body-related (43%) and mind-related appraisals (33%), while the less frequently mentioned appraisals were captured by the remaining four main categories mind-body-related (17%), daily-life-related (4%), knowledge-related (2%), and process- and context-related appraisals (1%).

 Analyzing frequencies of appraisals in the subcategories, results revealed that 60% of all Taiji teachers mentioned an “increase of internal balance and peace of mind” as a benefit Taiji-beginners may expect from regular Taiji practice. Other frequently mentioned benefits were “improvement of body awareness” (46%) and “improvement of physical functioning” (44%) such as breathing, circulation, blood pressure, immune system, digestion and sleep, “improvement of balance” (38%), “improvement of motor coordination” (37%), “increase of power of concentration” (32%), and “increase of flexibility” (32%). For complete results see Table 2.

#### 3.3.2. Quantitative Analyses

Taiji teachers appraised a moderate-to-high improvement of stress management in beginners as a result of regular Taiji practice (mean 54.36 ± 9.67; range = 12 to 72). As revealed in regression analysis, appraisal of a higher improvement of successful stress management in beginners was significantly predicted by lower teaching experience (*β* = − 0.22; *P* = 0.024; see Table 3). The overall explanation of variance by our model is small (*R* ^2^ = 0.075; *R* ^2^corr = 0.053; F = 3.46, df = 3/13, *P* = 0.018) with “teaching experience” alone explaining 3.7% (*P* = 0.024).

### 3.4. Explorative Comparison of Beginners' Expectations with Teachers' Appraisals

The comparison of the main categories of beginners' expectations and teachers' appraisals is depicted in Figure 1. In contrast to the Taiji teachers, beginners generally stated significantly fewer expectations (*t* = 6.14; df = 200, *P* < 0.001). They addressed significantly more frequently daily-life-related (*χ* ^2^ = 73.48; *P* < 0.001) and knowledge-related expectations (*χ* ^2^ = 104.61; *P* < 0.001) toward their upcoming Taiji course. Taiji teachers in turn were more frequently emphasizing body-related (*χ* ^2^ = 74.72; *P* < 0.001) and mind-related benefits (*χ* ^2^ = 27.06; *P* < 0.001) a Taiji-beginners course may offer. No group differences were observed for mind-body-related statements (*P* = 1.00). Process- and context-related statements were the least mentioned ones in both groups, yet significantly more often mentioned by Taiji-beginners (*χ* ^2^ = 62.48; *P* < 0.001). Both groups did not differ regarding their ratings related to expected, respectively, appraised Taiji-induced changes in overall stress management (*P* = 0.54).

## 4. Discussion

Our study is the first to examine beginners' expectations and teachers' appraisals towards Taiji-beginners courses. In the following, we will summarize, discuss, and compare our findings of our two surveys.

While both beginners and teachers expressed comparable mind-body-related expectations and appraisals, we found significant differences in daily-life-, knowledge-, mind- and body-related statements. Taiji-beginners expected to learn a new approach that is particularly helpful to foster peace of mind and to improve their stress management. They explicitly emphasized the transferability of course contents into their daily life. In contrast to Taiji-beginners, only a few teachers mentioned knowledge-related and daily-life-related benefits, but many of them stated mind- and body-related benefits of Taiji practice. It might be that Taiji teachers are mainly aiming to transmit implicit procedural rather than explicit declarative knowledge about Taiji to their students. As the retention of procedural knowledge is thought to be longer lasting [41], teachers might have implicitly assumed that the skills their students acquire during their Taiji training would have an impact on their daily life. On the other hand it should be kept in mind that the beginners under study took part in a research project that examined whether Taiji training-related to psychosocial stress reactivity [22]. This participation might have contributed to a frequent mentioning of improvement of stress management among beginners' daily-life-related expectations. However, since stress-management-related benefits of Taiji practice are commonly described in basic literature for Taiji-beginners [7, 42, 43], these expectations might also occur in subjects attending Taiji-beginners classes in naturalistic settings. A possible reason explaining why beginners did not mention body- and mind-related expectations as frequently as teachers did might be the lack of knowledge about these potential effects of Taiji practice. This reasoning is in line with the high frequency of knowledge related expectations in Taiji-beginners. Also to be taken into account is the young to middle age and the good health status of the beginners under study. While older people with an impaired physical condition are likely to expect more body-related benefits when engaging into physical activity programs [44] this might not have been the case for our study group. 

Only a few beginners and even fewer teachers mentioned process- and context-related expectations and appraisals. This finding suggests that the vast majority of our study participants are not aware of the potential relevance of process- and context-related factors for treatment outcome [45–48]. It may be that Taiji teachers take the agreeableness and the appropriateness of their teaching methods employed in their courses for granted and therefore rarely mention context and process related aspects of their work. For Taiji-beginners, it is very unlikely that they are already familiar with the special role of a Taiji-teacher in terms of being not only teacher but also motivator, coach and therapist [6] as they never experienced a Taiji-lesson before. 

Congruence of both study groups was observed in terms of moderate to high expected and appraised improvements in stress management in response to regular Taiji practice. Our quantitative data shows that a lower educational level predicted higher stress-management-related expectations in Taiji-beginners. This might be due to the fact that people with a less favorable educational background are affected more strongly by the presence of various stressors and absence of multiple resources [49] and therefore are more likely to express higher stress-management-related expectations. Personal resource factors such as mindfulness and self-compassion are regarded as stress protective trait characteristics [50, 51]. Therefore, it may be speculated, that subjects with low scores in these two variables have a higher need for improvements in stress management and that this need influences stress-management-related expectations. Notably, in this study we have also assessed self-reported mindfulness and self-compassion scores in all study participants at baseline examination [52]. Indeed, our explorative analysis revealed that there are significant negative correlations of the sum score of stress-management-related expectations with self-reported self-compassion total score (*r* = −0.27; *P* = 0.019), self-compassion subscales “isolation” (*r* = 0.34; *P* = 0.003), and “overidentification” (*r* = 0.30; *P* = 0.011) but not with mindfulness (*r* = −0.17; *P* = 0.15). Hence, our data partially support a potential association between lower levels of personal resource factors and a higher level of stress-management-related expectations. For the Taiji teachers interestingly, a shorter teaching experience predicted more optimistic teachers' appraisals regarding improvement in stress management. As more experienced teachers are believed to have larger teaching experience with advanced Taiji students, they therefore might perceive beneficial effects in long-term Taiji-practitioners as more pronounced than in beginners. Hence, our finding might be explained by a broader frame of reference employed by the more experienced Taiji teachers. 

 Our study results provide information with practical relevance. Considering the self-confirming nature of expected treatment responses [25–28], our data suggest that Taiji-beginners are likely to foster mental well-being and enhance stress management by implementing their acquired Taiji-related knowledge and skills in their everyday lives. In fact, we have observed decreased psychobiological reactivity to psychosocial stress [22] and enhanced levels of mindfulness and self-compassion [52] in subjects in the Taiji group compared to subjects in the waiting-list control group. However, as explorative analyses revealed, these effects have not been found to be directly modulated by stress-management related expectancies (data not shown). Potential synergistic effects of beginners' expectations with other treatment components such as teacher's appraisals on treatment outcomes should be subject of future research. Since beginners' expectations and teachers' appraisals differ in several points, we recommend Taiji teachers to proactively ask their new students about their course expectations and to inform them about potential benefits of regular Taiji practice early in the course. This can prevent students from disappointment due to clinging to inadequate expectancies and helps them to modify their expectations towards greater congruence with teachers' appraisals of potential benefits a Taiji beginner course may offer. Also teachers might adapt their courses to respond to their students' needs more effectively. Such congruence in turn would be likely to enhance the working alliance and to increase beginners' course satisfaction and course adherence. Despite this reasoning being highly plausible, our data do not allow to draw any conclusion about the impact of the observed incongruence between beginners' expectations and teachers' appraisals on the outcomes of the Taiji courses or the beginners' and/or teachers' course satisfaction. Still our findings may provide information of practical relevance: Since Taiji is not commonly practiced among health care professionals [53], our data collected from 136 Taiji teachers provides valuable insights into potential benefits of Taiji-beginners courses that might be helpful for health care professionals for their own as well as for their patients' information.

The following limitations need to be addressed. First, Taiji-beginners were not students of the investigated teachers; thus we were not able to assess the degree of working alliance between both study groups. For the same reason a potential negative influence of the mismatch between beginners' expectations and teachers' appraisals on course-related outcome values could not have been investigated. Secondly, the results of Taiji-beginners' expectations are restricted to healthy young to middle-aged and predominantly well-educated participants. People with functional limitations should be included in future studies, as this population is regarded to represent a large proportion of all Taiji practitioners [54]. Third, because our survey of Taiji-beginners was nested within a trial examining effects of Taiji on psychobiological stress reactivity, this circumstance might have influenced stress-management-related expectations of our Taiji novices. Therefore, we recommend for future studies in this field to investigate Taiji-beginners and their corresponding teachers under naturalistic conditions.

 In addition to the above-mentioned practical implications of our findings, the main strengths of this study are the consideration and comparison of both beginners' expectations as well as teachers' appraisals, the combination of qualitative and quantitative data assessment, which allows us to provide an overview of general as well as more specifically stress-management-related expectations and appraisals. Moreover, a relatively large population of active Taiji teachers has participated in our survey.

## 5. Conclusion

The results of our study increase the awareness of and knowledge about the nature of expectancies and appraisals in Taiji-beginners practice. We have found that educational background, the level of self-compassion, and teaching experience are involved in shaping stress-management-related expectations and appraisals. The impact of students' expectations, teachers' appraisals, and the interaction of both on treatment outcomes of Taiji interventions remains to be further investigated.

## Figures and Tables

**Table 1:** Socio-demographic data of study participants.

	Beginners (*n* = 74)	Teachers (*n* = 136)
Age in years (mean ± SD; range)	35.35 ± 7.49; 22–50	50.01 ± 8.46; 29–71
Gender		
Male (%)	30	49
Female (%)	70	51
Education level		
With high school degree (i.e., Swiss Matura) (%)	77	—
Without high school degree (%)	23	—
Occupation status		
Student (%)	11	0
Full- or part-time job (%)	89	100
Taiji practice in years (mean ± SD; range)	0	18.60 ± 8.26; 3–46
Taiji teaching practice in years (mean ± SD; range)	0	11.56 ± 7.75; 1–37

SD: standard deviation.

**Table 2:** Frequency of mentioned beginners' expectations and teachers' appraisals of the benefits of a Taiji-beginners course.

Mentioned expectations/appraisals	Beginners (*n* = 74) results in %^1^	Teachers (*n* = 136) results in %^1^
Daily-life-related expectations/appraisals		
Improvement of stress management	41	13
Transferability of course content into daily life	27	9
Counterbalance to daily work	14	3
Knowledge-related expectations/appraisals		
Get to know Taiji in general	57	2
Learning the motion sequences	15	4
Get to know the philosophical background	5	2
Improvement of self-defense	1	1
Mind-body-related expectations/appraisals		
Increase of body awareness	27	46
Increase of relaxation	26	29
Holistic health promotion	16	19
Perception of the flow of Qi/energy	1	8
Mind-related expectations/appraisals		
Increase of internal balance and peace of mind	32	60
Increase of power of concentration	14	32
Expansion of consciousness	5	6
Fostering of self-compassion	5	7
Fostering of equanimity	4	16
Increase of contentedness	3	16
Fostering of mindfulness	3	5
Increase of mental flexibility/openness	1	11
Increase of self-efficacy	1	5
Increase of patience and tenacity	1	1
Increase of self-esteem	0	10
Fostering of compassion and tolerance towards others	0	8
Increase of mental alertness	0	18
Improvement of memory	0	2
Body-related expectations/appraisals		
Be physically active	16	3
Increase of physical well-being	14	16
Strengthening of the body	8	21
Improvement of motor coordination	7	37
Increase of flexibility	5	32
Improvement of balance	4	38
Improvement of body alignment/posture	3	21
Improvement of physical functioning	3	44
Alleviation of physical ailments	1	23
Increase of postural stability	0	16
Increase of looseness	0	4
Reduction of risk of falls	0	1
Process- and context-related expectations/appraisals		
Enjoyment of practicing Taiji	12	5
Meeting new people	9	2
Professional instruction	9	0
Experience of learning progress	7	1
Pleasant course ambience	5	1

^1^% values refer to the percentage of subjects in each study group.

**Table 3:** Hierarchical regression analyses for (a) Taiji-beginners' expectations related to stress management and (b) for Taiji-teachers' appraisals related to stress management.

Variables entered	Standardized *β*-coefficient	*t *	*P* value	*R* ^2^ change
(a) Stress management (*n* = 74)				
Age	−0.16	−1.39	0.17	0.009
Gender	−0.08	−0.68	0.50	0.001
Education level*	−0.29	−2.48	0.016	0.080
(b) Stress management (*n* = 133)				
Age°	0.17	1.83	0.07	0.010
Gender°	0.14	1.68	0.10	0.027
Teaching experience*	−0.22	−2.28	0.024	0.037

°*P* ≤ 0.10; *P ≤ 0.05.

## Appendix

### Questionnaire Assessing Expected/Perceived Changes in Stress Management Attributed to Taiji Practice

Please indicate to which degree the below mentioned statements match your personal opinion: 1 = strongly disagree; 2 = disagree; 3 = rather disagree; 4 = rather agree; 5 = agree; 6 = strongly agree.

- Items answered by Taiji-beginners: From regular Taiji practice, *I do expect that I can…* *
- Items answered by Taiji teachers: A beginner who is regularly practicing Taiji* can expect that he/she can…
  1. deal with stressful situations in a more relaxed manner;
  2. be more self-aware in difficult situations;
  3. increase my, resp., his/her feeling of physical fitness;
  4. better perceive the needs of my, resp., his/her body;
  5. generally feel more calm and balanced;
  6. be more mindful in daily life;
  7. feel less troubled by unexpected inconveniences;
  8. rely more on my, resp., his/her inner strengths when facing unexpected inconveniences;
  9. maintain calmness in challenging situations;
  10. recover more rapidly after a demanding task;
  11. increase my, resp., his/her power of concentration;
  12. socialize more with others.

*Regular class attendance twice a week during three months including independent Taiji practice at home.
